# Using Supervised Principal Components Analysis to Assess Multiple Pollutant Effects

**DOI:** 10.1289/ehp.9226

**Published:** 2006-08-24

**Authors:** Steven Roberts, Michael A. Martin

**Affiliations:** School of Finance and Applied Statistics, College of Business and Economics, Australian National University, Canberra, Australian Capital Territory, Australia

**Keywords:** air pollution, mortality, multiple pollutants, principal components analysis, time series

## Abstract

**Background:**

Many investigations of the adverse health effects of multiple air pollutants analyze the time series involved by simultaneously entering the multiple pollutants into a Poisson log-linear model. This method can yield unstable parameter estimates when the pollutants involved suffer high intercorrelation; therefore, traditional approaches to dealing with multicollinearity, such as principal component analysis (PCA), have been promoted in this context.

**Objectives:**

A characteristic of PCA is that its construction does not consider the relationship between the covariates and the adverse health outcomes. A refined version of PCA, supervised principal components analysis (SPCA), is proposed that specifically addresses this issue.

**Methods:**

Models controlling for long-term trends and weather effects were used in conjunction with each SPCA and PCA to estimate the association between multiple air pollutants and mortality for U.S. cities. The methods were compared further via a simulation study.

**Results:**

Simulation studies demonstrated that SPCA, unlike PCA, was successful in identifying the correct subset of multiple pollutants associated with mortality. Because of this property, SPCA and PCA returned different estimates for the relationship between air pollution and mortality.

**Conclusions:**

Although a number of methods for assessing the effects of multiple pollutants have been proposed, such methods can falter in the presence of high correlation among pollutants. Both PCA and SPCA address this issue. By allowing the exclusion of pollutants that are not associated with the adverse health outcomes from the mixture of pollutants selected, SPCA offers a critical improvement over PCA.

Numerous time-series studies have investigated the association between daily adverse health outcomes and daily ambient air pollution concentrations (Chock et al. 2000; Cifuentes et al. 2000; Goldberg et al. 2003; Kelsall et al. 1997; Kwon et al. 2001; Moolgavkar 2000; Ostro et al. 1999; Smith et al. 2000; Stieb et al. 2002). These studies typically fit a Poisson log-linear model to concurrent time series of daily mortality or morbidity, ambient air pollution, and meteorologic covariates. The fitted models are then used to quantify the adverse health effects of ambient air pollution. Because the U.S. Environmental Protection Agency regulates pollutants independently, much of the current time-series research on the adverse health effects of air pollution has focused on estimating the effect of an individual pollutant (Dominici and Burnett 2003). However, because of the potential for high correlations to exist between ambient air pollutants, the results from studies that focus on a single pollutant can be difficult to interpret in practice (Vedal et al. 2003). For example, an observed positive association could occur because the single air pollutant is a proxy for another air pollutant or for a mixture of air pollutants.

To overcome the limitations of single-pollutant time-series studies, a number of studies have investigated the concurrent adverse health effects of multiple air pollutants (Moolgavkar 2000; Wong et al. 2002). In the majority of these studies, the multiple air pollutants are simultaneously entered into a single Poisson log-linear model. The results from these studies are then used to isolate the adverse health effects of the individual pollutants. However, one important question that these multiple pollutant studies fail to answer is whether there is a specific mixture of pollutants associated with adverse health outcomes. Moreover, it has recently been stated that it may be more reasonable to assume that there is a mixture of pollutants that is considered harmful to health (Dominici and Burnett 2003; Moolgavkar 2003; Stieb et al. 2002). Assessing the adverse health effects of an air pollution mix may therefore be both more interpretable and more feasible than attempting to isolate the effects of individual pollutants independent of other pollutants. The development of new methodology and models to concurrently estimate the adverse health effects of multiple air pollutants has been identified by statisticians, epidemiologists, and policymakers as an important area of ongoing research (Cox 2000; Dominici and Burnett 2003). A number of studies have addressed this issue using methods ranging from the calculation of air pollution indices to the application of shrinkage-based methods such as ridge regression and the lasso (Hong et al. 1999; Roberts and Martin 2005, 2006a).

One method used or proposed by researchers to analyze the effect of multiple pollutants is principal components analysis (PCA) (Burnett et al. 2003; Cox 2000). PCA avoids the problem of unstable parameter estimates sometimes obtained in multiple pollutant studies because of the high correlations between pollutants. However, one characteristic of PCA in this context is that the mixture of pollutants identified as a principal component is constructed using only covariate information without regard to the relationship between pollutant levels and mortality. We investigate a recently proposed modified version of PCA called supervised principal component analysis (SPCA) for analyzing the adverse health effects of multiple pollutants. SPCA was developed by Bair et al. (2006) for use in regression problems in which the number of predictors greatly exceeds the number of observations. In this article we refine their implementation of SPCA to make it suitable for use in multiple pollutant studies. For this purpose SPCA is similar to conventional PCA except that it uses a subset of the multiple pollutants that are selected on the basis of their association with the adverse health outcomes of interest rather than only on intrinsic properties of the covariate space. As a result, SPCA is allowed to exclude pollutants not associated with the adverse health outcomes from the mixture of pollutants it returns.

In addition to PCA, a number of methods have been developed in the regression literature to deal with the problem of high correlations among covariates or predictor variables. These methods include ridge regression, partial least squares, and latent root regression (Bertrand et al. 2001). Similar to SPCA, partial least squares and latent root regression use information in the response variable to construct latent variables (linear combinations of the predictor variables) to be used as predictors. Latent root regression shares another feature in common with SPCA in that it also makes use of a PCA to construct the latent variables. Latent root regression and partial least squares may also prove useful tools in assessing the adverse health effects of multiple air pollutants. For this reason future studies that investigate the use of these methods for assessing the adverse health effects of multiple air pollutants may prove valuable. Further information on latent root regression can be found in Gunst et al. (1976) and Webster et al. (1974), and further information on partial least squares can be found in Hoskuldsson (1988).

## Materials and Methods

### Materials

The data used in this article were obtained from the publicly available National Morbidity, Mortality, and Air Pollution Study (NMMAPS) database (Johns Hopkins Bloomberg School of Public Health 2005). The data extracted consists of concurrent daily time series of mortality, weather, and air pollution for nine cities in the United States from 1987 to 2000. The nine cities selected had a relatively large number of days with measurements for all five air pollutants considered. Many of the cities in the NMMAPS database do not collect data on all five air pollutants and/or have a large number of days with missing air pollutant concentrations. Further details on the data used can be obtained at http://www.ihapss.jhsph.edu/ (Johns Hopkins Bloomberg School of Public Health 2005).

The mortality time-series data, aggregated at the county level, are nonaccidental daily deaths of individuals 65 years of age and older. Deaths of nonresidents were excluded from the mortality counts. The weather time-series data are 24-hr averages of temperature and dew-point temperature, computed from hourly observations. The five air pollutants considered are particulate matter (PM) of <10 μm in diameter (PM_10_), ozone, sulfur dioxide, carbon monoxide, and nitrogen dioxide. For PM_10_, SO_2_, CO, and NO_2_, average daily concentrations were used. For O_3_ the maximum hourly concentration for each day was used. In the analyses that follow, each of the pollutant time series was standardized to have unit variance.

### Methods

The majority of time-series studies that have investigated the concurrent adverse health effects of multiple air pollutants simultaneously entered the pollutants into a single Poisson log-linear model. With this model the daily adverse health outcome counts are modeled as independent Poisson random variables with a time-varying mean μ*_t_*, where


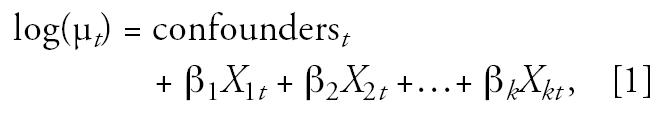


and where confounders*_t_* represents other time-varying variables related to the adverse health outcomes, *X_it_*, *i* = 1,..,*k* represent the *k* pollutants under investigation, and β*_i_*, *i* = 1,..,*k*, measure the adverse health effect of pollutant *i*, assuming all other pollutant levels are held fixed. Hereafter, Model 1 will be referred to as the “standard model.”

A noted problem with the standard model is that the pollutant-effect parameter estimates may be unstable (i.e., have unduly high covariance structure) because of high correlation among pollutants (Burnett et al. 2003). When high correlation exists among pollutants, one of the pollutants can be well approximated by a linear combination of the remaining pollutants, resulting in the fitted model becoming close to unidentifiable, and the associated parameter estimates becoming unstable (Ramsay et al. 2003). In the context of linear regression, this is commonly referred to as the problem of multicollinearity. When generalized linear models (GLMs) or generalized additive models (GAMs) are used, an analogous problem to multicollinearity, concurvity, can occur. Concurvity refers to the situation in which a function of one of the covariates is well approximated by a linear combination of the functions of other covariates. In our context the functions would be the smooth functions used to model the effects of the confounding covariates. Like multicollinearity, concurvity results in unstable parameter estimates from the fitted model. In the presence of concurvity, it was noted by Ramsay et al. (2003) and Dominici et al. (2002) that the variance estimates obtained from a fitted GAM do not reflect the resulting instability of the parameter estimates. To avoid this problem, Ramsay et al. (2003) suggested that the GLM be used instead of the GAM and that the confounding covariates be modeled parametrically, for example, by using natural cubic splines. For this reason all models in this article will be posed as the GLM with natural cubic splines used to model the effect of the confounding variables. This is the same modeling approach that has been adopted in a number of recent studies (Luginaah et al. 2005; Peng et al. 2005).

PCA is a method commonly used in regression analysis to overcome the problems associated with correlated explanatory variables. In the context of the standard model, PCA finds the linear combination of the pollutant variables that has maximal variance among all such combinations. Specifically, constants α*_i_*, *i* = 1,..,*k* are found such that the variance of *Z*_1_*_t_* = α_1_*X*_1_*_t_* + α_2_*X*_2_*_t_* + … + α*_k_X_kt_* is maximized. The standard model is then refit using the derived variable *Z*_1_*_t_*, referred to as the first principal component, in place of the *k* original pollutant variables *X_it_*, *i* = 1,..,*k*:


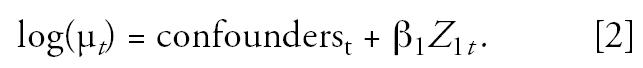


Using the single derived variable Z_1_*_t_* in Model 2 avoids the coefficient instability problems associated with fitting a model to the correlated pollutant variables. A simple justification for PCA is that by choosing the linear combination with maximum variance we are retaining as much of the information contained in *X_it_*, *i* = 1,..,*k* as possible with the use of only a single variable *Z*_1_*_t_*. It should be noted that a complete PCA of the pollutant data returns *k* independent principal components *Z_it_*, *i* = 1,..,*k*, each describing successively less of the information (variance) contained in *X_it_*, *i* = 1,..,*k*. In many cases the first one or two principal components capture almost all the variability in the covariate space, and so for ease of interpretation we elect here to consider only the first and most important PCA variable in our analysis. Hereafter, Model 2 will be referred to as the “PCA model.”

One characteristic of PCA is that the pollutant mixture *Z*_1_*_t_* = α_1_*X*_1_*_t_* + α_2_*X*_2_*_t_* +…+ α*_k_X_kt_* derived from the PCA model is chosen without regard to the response variable—the daily adverse health outcomes of interest. The PCA approach was designed to reduce the dimension of a high-dimensional covariate space so that fewer variables might be considered in an analysis. As such, the relationship between the covariates and the response is simply not considered in constructing the principal components. This is a potentially undesirable characteristic because pollutants that are not associated or only weakly associated with the adverse health outcomes will, all other things being equal, be treated exactly the same by PCA as those pollutants strongly associated with the adverse health outcomes. SPCA is a modification of PCA that avoids this characteristic by explicitly incorporating information on the relationship between the predictor variables and the response. It should be noted that SPCA shares a feature similar to another multivariate analysis technique, canonical correlation analysis, that also finds linear combinations of the predictor variables that depend on the response variable or variables. However, the two methods have important differences, including the criterion used for finding the optimal linear combinations and that SPCA is used when there is a single response variable and a number of explanatory variables, whereas canonical correlation analysis is used when there are a number of response variables and a number of explanatory variables.

As originally proposed, SPCA was designed for regression problems in which the number of predictors greatly exceeds the number of observations. Here we implement a version of SPCA that can be used as an alternative to PCA in multiple pollutant studies, a situation where the number of observations generally exceeds the number of parameters. Our implementation of SPCA proceeded as follows:

1. Fit a separate Poisson log-linear model for each pollutant variable, relating the confounders and the given pollutant to the adverse health outcomes. That is, for each pollutant (*X_it_*, *i* = 1,..,*k*), fit the model log(μ*_t_*) = confounders*_t_* + β_1_*X_it_*.
2. For each of the *k* models fit in step 1, note the absolute value of Wald’s statistic *w* = |*b*_1_/SE(*b*_1_)|]. Order the pollutant variables from most important to least important based on decreasing values of *w*. Denote this ordered list of pollutant variables *X*_[_*_i_*_]_,*i* = 1,..,*k*, where X_[_*_i_*_]_ corresponds to the pollutant with the *i*^th^ largest value of *w*.
3. Using the first 90% of the data, fit the following *k*+1 models: log(μ*_t_*) = confounders*_t_* +β_1_*Q_it_*, *i* = 0,1,..,*k*, where *Q_it_*, *i* = 1,..,*k* corresponds to the first principal component of the pollutants *X*_[1]_,..,*X*_[_*_k_*_]_ and *Q*_0_*_t_* = 0, which corresponds to fitting a model with no pollutants, i.e., the model log(μ*_t_*) = confounders*_t_*.
4. Using the remaining 10% of the data, calculate the prediction error for each of these *k*+1 models, and select as the “best” of these models that with the smallest estimated prediction error.
5. Refit the best model using all the data, that is, fit the model log(μ*_t_*) = confounders*_t_* + β_1_*Q_st_*, where *s* corresponds to the best model.

Step 3 in the above algorithm could be implemented using more than the first principal component variable. However, for the reasons discussed in our implementation of the PCA model, we have decided to use only the first principal component variable. The advantage of the SPCA procedure over PCA is that pollutants not associated or only weakly associated with the adverse health outcomes have a significant chance of being excluded from the chosen model. Hereafter, our implementation of SPCA using the above algorithm will be referred to as the “SPCA model.”

## Simulation Study

To conduct the simulations, we required a way of generating realistic mortality time series with known air pollution mortality effects. We used a method previously shown to generate realistic mortality time series (Roberts and Martin 2006b), which proceeds by fitting the following Poisson log-linear model similar to those used in previous analyses (Daniels et al. 2000), to the actual Cook County (Chicago), Illinois, mortality and meteorologic time-series data:


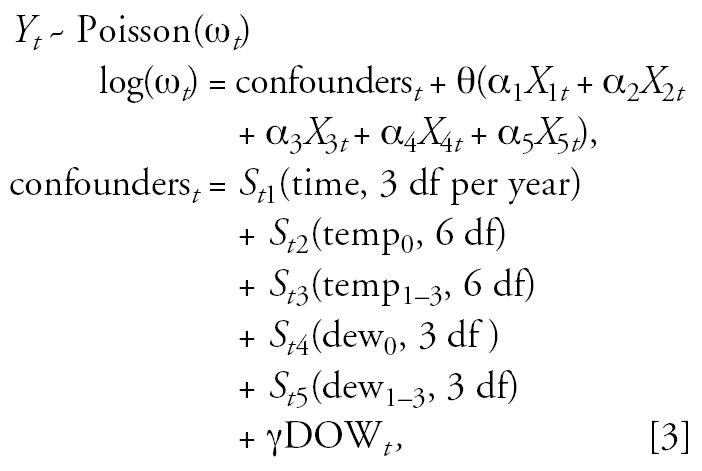


where the *t* refers to the day of the study, *Y_t_* is the simulated mortality count on day *t* and ω*_t_* is the expected number of deaths on day *t*. The quantities *S_ti_*() are smooth functions of time, temperature (temp), and dew-point temperature (dew) with the indicated degrees of freedom (df). The smooth functions are represented using natural cubic splines. The quantity temp_0_ is the current day’s mean 24-hr temperature and temp_1–3_ is the average of the previous 3 days’ 24-hr mean temperatures. The values dew_0_ and dew_1–3_ are defined similarly for the 24-hr mean dew-point temperature, and DOW*_t_* is a set of indicator variables for the day of the week. The quantities *X_it_*, *i* = 1,..,5, are, respectively, the current day’s daily concentrations of PM_10_, NO_2_, CO, O_3_, and SO_2_, α*_i_*, *i* = 1,..,5, are the prespecified weights of each pollutant, and θ is the prespecified effect of the air pollutant mixture (α_1_*X*_1_*_t_* +…+ α _5_ *X*_5_*_t_*) on mortality. All the analyses in this article were conducted using the statistical package R (R Development Core Team 2006). For the reasons discussed above, the GLMs along with natural cubic splines were used to fit the PCA and SPCA models. The offset option in the R GLM function was used to permit the relationship between the pollutants and mortality to be *a priori* specified and included in the fitting process.

Fitting Model 3 produced an expected mortality count for each day, ω*_t_*, that incorporated the effects of the five pollutants through the explicitly specified relationship θ (α_1_*X*_1_*_t_* +…+ α_5_*X*_5_*_t_*). Using these expected mortality counts, the simulations proceeded as follows:

1. Choose values for (θ, α_1_, α_2_, α_3_, α_4_, α_5_).
2. Using Model 3, compute expected mortality counts for each day, ω*_t_*, that incorporate the pollutant effects selected in step 1.
3. Generate a mortality time series using a Poisson model with mean ω*_t_* on day *t*.
4. Fit the PCA model to the simulated mortality time series; that is, fit the model log(μ*_t_*) = confounders*_t_* + β_1_*Z*_1_*_t_* to the simulated mortality time series from step 3.
5. Fit the SPCA model to the simulated mortality time series, that is, using the simulated mortality time series from step 3, follow steps 1–5 in the description of the SPCA model. This procedure results in a final model of the form log(μ*_t_*) = confounders*_t_* + β_1_*Q_st_* being fitted to the simulated mortality time series.
6. Repeat steps 3–5 1,000 times.

In the simulations, 13 (θ, α_1_, α_2_, α_3_, α_4_,α _5_) combinations were used. For these 13 combinations, the effect of the air pollutant mixture on mortality θ ranged from 0 to 0.1. A θ value of 0.1 corresponds to approximately a 10% increase in mortality for a simultaneous one standard deviation (SD) increment in the concentration of each air pollutant. Tables 1–3 contain the results of the simulations.

Table 1 shows clearly that in most situations SPCA performed appropriately in terms of selecting the correct subset of pollutants associated with mortality. In 9 of the 13 scenarios considered, SPCA selected the correct subset of pollutants 75% or more of the time. This is an important improvement over standard PCA, which by construction will never exclude pollutants. Table 2 shows that for the majority of cases considered, the bias of the individual pollutant effect estimate obtained from SPCA was smaller than the bias of the corresponding estimate obtained from PCA. The reduction in bias was particularly striking for cases in which an individual pollutant was unrelated to mortality. The smaller bias of the SPCA estimates compared to the PCA estimates is because pollutants unrelated to mortality are retained by PCA; these unrelated pollutants explain by chance some of the morality effect that should be attributed to pollutants that are actually associated with mortality. However, the ability of SPCA to exclude pollutants is not without cost. The ability of SPCA to exclude pollutants means that the weights it assigns to each pollutant in the derived pollutant variable (*Q_st_*) are random, unlike the constant weights assigned by PCA. The random nature of the SPCA weights will typically result in the estimates obtained from SPCA having a larger variance than the corresponding estimates obtained from PCA. The increased variance is evidenced in Table 2, where for the majority of cases considered, the SD of the individual pollutant effect estimate obtained from SPCA is larger than the SD of the corresponding estimate obtained from PCA.

The root-mean-squared error (rmse) is a measure of the average “closeness” of an estimator to the value that is being estimated; smaller values of the rmse correspond to “better” estimators. The rmse values in Table 3 indicate that SPCA produced better estimates or estimates with smaller error in slightly more than half of the cases considered. For these cases the increased variance of the SPCA estimates was more than compensated for by a reduction in bias. Perhaps more important, the rmse values for SPCA were much more stable under different simulation scenarios than the rmse values for PCA; the rmse values for SPCA ranged from 0 to 12.06 with a median of 1.71, whereas the rmse values for PCA ranged from 0.33 to 80.99 with a median of 2.58. This tells us that the benefits of using SPCA instead of PCA, in terms of both bias and the average closeness of the associated estimates to their true values, outweigh the disadvantage in terms of variance alone of using SPCA instead of PCA. This suggests that aside from providing useful information on which pollutants are unrelated to mortality that the SPCA model has the additional benefit over the PCA model of producing estimates with smaller error on average.

## Application

In this section the data from the nine cities described previously are used to illustrate the use of the SPCA model compared to the PCA model in the multiple pollutant context. For both models, the confounder adjustments used had the same specification described in the previous section for Model 3. Table 4 contains the results of fitting the models to the data from each city. Table 5 provides correlation matrices of the data from two of the nine cities considered—Cleveland, Ohio, and Nashville, Tennessee. The correlation matrices for these two cities are shown because they correspond to the two cities where the SPCA model retained the least (zero pollutants) and most (four pollutants) pollutants, respectively.

The results obtained from the two methods differ substantially, as for each city SPCA concluded that one or more of the five pollutants were not sufficiently associated with mortality to warrant inclusion. This in turn results in the two methods returning different estimates for the effect of air pollution on mortality. Since the results of the simulation study suggested that SPCA was successful in the majority of cases in determining the correct subset but not necessarily the magnitude of pollutants associated with mortality, the results obtained with SPCA are likely more reliable than those obtained with PCA. For example, for the Chicago data, SPCA concluded that only O_3_ was associated with mortality and gave it a loading of one, while PCA gave each pollutant a roughly equal loading. In this situation the effect estimate obtained using PCA is likely biased because the mixture of pollutants on which this estimate is based contains a number of pollutants that may be unrelated to mortality.

The results from the SPCA model reveal interesting interpretations from the analysis about the effects of the five air pollutants on mortality in the nine cities considered, specifically highlighting when a particular pollutant appears unrelated to mortality. This insight is unavailable from the results of the PCA model, which invariably implicates all five pollutants albeit with differing weights. In all cities SPCA suggests that the pollutant mixture associated with daily mortality consists of only a subset of the five pollutants considered. Additionally, in Cleveland, Ohio; Houston, Texas; and Salt Lake City, Utah, none of the pollutants was found to be associated with mortality. This additional information could prove valuable to researchers interested in determining the specific pollutants associated with increased mortality in particular regions. Of course, a question of considerable interest raised by the SPCA results is why a particular pollutant, for example, PM_10_, is found to be associated with mortality in some cities but not in others.

The correlation matrices in Table 5 suggest reasons for the weights or loadings assigned to each pollutant by the PCA and SPCA models. In Cleveland the five pollutants considered are all interrelated to roughly the same extent, which results in the first PCA variable, or first derived variable (as seen in Table 4), giving each pollutant roughly equal loading or weight. In Cleveland, SPCA did not retain any pollutants, but the correlation structure of the pollutants for this city indicates that, like PCA, any pollutants retained by SPCA would have received a roughly equal loading. However, in Nashville because SO_2_ is essentially unrelated to the other pollutants, it is given a relatively small weight in both the first derived SPCA and PCA variables.

## Discussion

Principal component analysis is a commonly used remedial measure for multicollinearity. The generally high positive correlation that exists between ambient air pollutants makes PCA a useful tool for multiple pollutant time-series studies. However, the use of PCA for this purpose raises concerns, of which the primary concern is that PCA invariably includes all pollutants in the selected mixture of air pollutants. A modified version of SPCA was shown to successfully deal with this problem, allowing a subset of the pollutants to contribute to a mixture related to the adverse health outcomes of interest. A shortcoming of SPCA, like PCA, is that once SPCA has selected the appropriate subset of pollutants, the loadings applied to each pollutant in the subset are also assigned without regard to the adverse health outcomes of interest. An interesting article by Hadi and Lin (1998) provides further cautionary notes on the use of PCA, many of which are also applicable to SPCA.

In this article we have considered only the implementation of PCA and SPCA using the first derived variable, that is, the first principal component of all five pollutants for PCA and the first principal component of the retained pollutants for SPCA. We made this choice because for the pollutant data used in this article, the first derived variable captured a significant proportion of the variability and using only a single derived variable provides a single linear combination of pollutants to be interpreted. This simplification also allowed a more concise description of the new methodology. If more than one derived variable is used, it will be necessary to interpret other linear combinations of pollutants, combinations that often do not have an intuitive interpretation and that explain a relatively small proportion of overall variability. Of course, both the PCA and SPCA methods can be extended to consider more than a single derived variable. Indeed, the inclusion of additional derived pollutant variables in either or both PCA and SPCA may result in improved performance in terms of the bias and variance properties of the resulting pollutant effect estimates. For example, if the first derived variable in a PCA gives large weights to pollutants unrelated to mortality compared with those actually associated with mortality, then including additional derived variables may result in improved estimates. However, for reasons of brevity and clarity, we elected to consider only the first derived variable in each case in describing the proposed methodology.

A number of different methodologies have been used to investigate the mixture of pollutants associated with an adverse health outcome. Hong et al. (1999) used a number of air pollution indices to evaluate the combined effects of various air pollutants. The indices used by Hong et al. were selected *a priori* and gave each pollutant included in the air pollutant index equal weight. This method is similar to using the first PCA of the multiple pollutants to estimate the adverse health effects of the multiple pollutants. The Hong et al. (1999) method possibly could be improved by using the same methodology employed in SPCA to remove unrelated pollutants. Other articles have investigated the mixture of pollutants associated with adverse health outcomes by assigning weights to each air pollutant that were explicitly estimated during the fitting process and constrained to sum to one (Roberts 2006a, 2006b). These weighted methods have benefits over both PCA and SPCA in that the loadings assigned to each pollutant depend on the adverse health outcomes. The disadvantage of the weighted methods is that they do not avoid the problem of unstable parameter estimates that can arise because of the positive correlation among pollutants. Each of these methods has merit, but in each case a key factor in whether the method appropriately deals with individual pollutants is the extent to which the pollutants are correlated with one another. In the presence of high intercorrelation among the pollutants, SPCA offers a critical advantage over these techniques. Another recent study investigated the use of the “shrinkage methods” ridge regression and the lasso for use in assessing the adverse health effects of multiple pollutants (Roberts and Martin 2005). Ridge regression and the lasso are methods that can be applied in a regression setting when some predictor variables are highly correlated. Again, these two methods have advantages over both PCA and SPCA in that the loadings assigned to each pollutant are dependent on the adverse health outcomes. However, an important advantage SPCA has over these shrinkage methods is that it is often able to successfully select the correct subset of pollutants that are associated with mortality. SPCA can be considered a method positioned somewhere between the shrinkage-based methods and the weighted methods. Like the shrinkage methods, SPCA is able to avoid unstable parameter estimates due to multicollinearity, and like the weighted methods it is often able to successfully select the correct subset of pollutants that are associated with mortality.

## Figures and Tables

**Table 1:** Number of pollutants retained by SPCA over sets of 1,000 simulations.

	No. of pollutants retained by SPCA^b^						
Effect (1,000 × θ)^a^	0	1	2	3	4	5	Percent correct^c^
All pollutants associated with mortality (α_1_ = α_2_ = α_3_ = α_4_ = α_5_= 1/5)							
100	0	0	0	15	0	85	85
50	0	0	1	24	0	75	75
25	0	3	8	22	3	65	65
12.5	0	11	11	16	8	54	54
PM_10_, NO_2_, and CO associated with mortality (α_1_ = α_2_ = α_3_ = 1/3,α_4_ = α_5_ = 0)							
100	0	0	0	100	0	0	100
50	0	0	0	100	0	0	100
25	0	2	6	86	4	3	86
12.5	0	11	14	45	12	18	44
PM_10_ associated with mortality (α_1_ = 1,α_2_ = α_3_ = α_4_ = α_5_ = 0)							
100	0	100	0	0	0	0	100
50	0	100	0	0	0	0	100
25	0	100	0	0	0	0	100
12.5	0	94	5	1	0	0	94
No pollutant associated with mortality (α_1_ = α_2_ = α_3_ = α_4_ = α_5_= 0)							
0	30	27	16	9	8	10	30

a 1,000× the actual values of θ used to generate mortality.

b The percentage of time over each set of 1,000 simulations that a subset of pollutants of a particular size was retained by SPCA.

c The percentage of time over each set of simulations that SPCA retained the correct subset of pollutants. The corresponding values for PCA will be 100% for the cases in which all pollutants were associated with mortality and 0% for all other cases.

**Table 2:** Bias and SD of the individual pollutant effect estimates obtained from SPCA and PCA over sets of 1,000 simulations.

	PM_10_		NO_2_		CO		O_3_		SO_2_	
Efffect (1,000 × θ) a	Bias^b^	SD^c^	Bias	SD	Bias	SD	Bias	SD	Bias	SD
All pollutants associated with mortality (α_1_= α_2_ = α_3_ = α_4_ = α_5_= 1/5)										
100	−0.09 (−0.50)	1.08 (0.53)	4.28 (3.48)	1.95 (0.64)	0 (−1.04)	2.47 (0.51)	−11.51 (−9.98)	3.62 (0.27)	−3.44 (−0.47)	7.06 (0.53)
50	0.46 (−0.09)	1.60 (0.53)	2.71 (1.94)	1.73 (0.64)	0.36 (−0.36)	2.13 (0.51)	−6.20 (−4.91)	2.23 (0.27)	−2.60 (−0.07)	4.35 (0.53)
25	1.18 (0.13)	2.47 (0.55)	1.63 (1.17)	2.03 (0.66)	0.05 (−0.02)	2.07 (0.53)	−3.15 (−2.37)	1.39 (0.28)	−1.67 (0.13)	2.50 (0.55)
12.5	1.55 (0.24)	2.63 (0.53)	0.80 (0.81)	1.72 (0.64)	−0.01 (0.17)	1.59 (0.52)	−1.34 (−1.09)	1.08 (0.27)	−0.90 (0.25)	1.46 (0.53)
PM, NO_2_, and CO associated with mortality (α_1_ = α_2_ = α_3_= 1/3,α_4_ = α_5_ = 0)										
100	−4.78 (−9.95)	0.62 (0.53)	3.61 (−5.18)	0.80 (0.64)	−0.19 (−10.6)	0.72 (0.52)	0 (12.01)	0 (0.27)	0 (23.4)	0 (0.53)
50	−2.06 (−4.84)	0.83 (0.51)	2.18 (−2.43)	1.01 (0.62)	0.22 (−5.17)	1.06 (0.50)	0 (6.07)	0 (0.26)	0 (11.8)	0 (0.51)
25	−0.23 (−2.27)	2.09 (0.54)	1.24 (−1.03)	2.08 (0.65)	−0.02 (−2.43)	2.28 (0.52)	0.26 (3.12)	1.03 (0.28)	0.17 (6.08)	1.01 (0.54)
12.5	1.18 (−0.98)	2.87 (0.55)	0.25 (−0.33)	2.26 (0.66)	−0.52 (−1.07)	2.15 (0.53)	0.65 (1.64)	1.04 (0.28)	0.57 (3.19)	1.25 (0.55)
PM associated with mortality (α_1_ = 1,α_2_ = α_3_ = α_4_ = α_5_ = 0)										
100	−1.76 (−80.99)	1.62 (0.52)	0 (22.90)	0 (0.62)	0 (18.50)	0 (0.50)	0 (9.77)	0 (0.27)	0 (19.1)	0 (0.52)
50	0.61 (−40.51)	1.77 (0.52)	0 (11.43)	0 (0.63)	0 (9.23)	0 (0.51)	0 (4.87)	0 (0.27)	0 (9.5)	0 (0.52)
25	1.99 (−20.12)	1.84 (0.52)	0 (5.88)	0 (0.63)	0 (4.75)	0 (0.51)	0.05 (2.51)	0.97 (0.27)	0 (4.89)	0 (0.52)
12.5	2.29 (−9.90)	2.31 (0.55)	0.03 (3.13)	0.45 (0.66)	0.02 (2.52)	0.32 (0.54)	0.50 (1.33)	2.10 (0.28)	0 (2.6)	0 (0.55)
No pollutant associated with mortality (α_1_= α_2_ = α_3_ = α_4_= α_5_= 0)										
0	0.80 (0.34)	1.62 (0.54)	0.29 (0.41)	0.75 (0.64)	0.38 (0.33)	0.87 (0.52)	0.14 (0.18)	0.90 (0.27)	−0.10 (0.34)	1.03 (0.54)

a 1,000× the actual values of θ used to generate mortality.

b 1,000× the bias of the estimated individual pollutant effects obtained from SPCA. The bias for PCA appears in parentheses.

c 1,000× the SD of the estimated individual pollutant effects obtained from SPCA. The SD for PCA appears in parentheses.

**Table 3:** Root-mean-squared error of the individual pollutant effect estimates obtained from SPCA and PCA over sets of 1,000 simulations.

Effect (1,000 × θ)^a^	PM_10_	NO_2_	CO	O_3_	SO_2_
All pollutants associated with mortality (α_1_ = α_2_ = α_3_ = α_4_ =α_5_ = 1/5)					
100	1.08 (0.73)^b^	4.70 (3.54)	2.46 (1.16)	12.06 (9.98)	7.85 (0.71)
50	1.66 (0.54)	3.22 (2.04)	2.16 (0.63)	6.59 (4.91)	5.06 (0.53)
25	2.73 (0.56)	2.60 (1.35)	2.07 (0.53)	3.44 (2.38)	3.01 (0.57)
12.5	3.05 (0.58)	1.90 (1.03)	1.58 (0.54)	1.71 (1.12)	1.72 (0.59)
PM, NO_2_, and CO associated with mortality (α_1_ = α_2_ = α_3_ = 1/3, α_4_ = α_5_ = 0)					
100	4.82 (9.97)	3.70 (5.22)	0.74 (10.61)	0 (12.02)	0 (23.43)
50	2.23 (4.87)	2.40 (2.5)	1.08 (5.19)	0 (6.08)	0 (11.86)
25	2.10 (2.33)	2.42 (1.21)	2.28 (2.49)	1.06 (3.13)	1.02 (6.10)
12.5	3.10 (1.12)	2.28 (0.74)	2.21 (1.19)	1.23 (1.66)	1.37 (3.24)
PM associated with mortality (α_1_ = 1,α_2_ = α_3_ = α_4_ = α_5_ = 0)					
100	2.39 (80.99)	0 (22.91)	0 (18.50)	0 (9.77)	0 (19.06)
50	1.88 (40.52)	0 (11.44)	0 (9.24)	0 (4.88)	0 (9.52)
25	2.71 (20.12)	0 (5.92)	0 (4.78)	0.97 (2.52)	0 (4.92)
12.5	3.25 (9.92)	0.45 (3.20)	0.32 (2.58)	2.16 (1.36)	0 (2.66)
No pollutant associated with mortality (α_1_ = α_2_ = α_3_ = α_4_ = α_5_ = 0)					
0	1.80 (0.63)	0.80 (0.76)	0.95 (0.62)	0.91 (0.33)	1.04 (0.64)

a 1,000× the actual values of θ used to generate mortality.

b 1,000× the root-mean-squared error of the estimated individual pollutant effects obtained from SPCA. The root-mean-squared error for PCA appears in parentheses.

**Table 4:** Results of fitting PCA and SPCA to the data from nine U.S. cities for 1987–2000.

	Pollutant loadings^a^					
City	PM_10_	NO_2_	CO	O_3_	SO_2_	Total effect^b^
Chicago, IL						
SPCA	0	0	0	1	0	0.005 (0.003)
PCA	0.466	0.556	0.45	0.231	0.467	−0.001 (0.003)
Cleveland, OH						
SPCA	0	0	0	0	0	0 (0)
PCA	0.496	0.512	0.437	0.351	0.42	−0.002 (0.006)
Denver, CO						
SPCA	0	−0.645	−0.678	0.353	0	0.013 (0.004)
PCA	0.484	0.515	0.536	−0.155	0.435	0.014 (0.006)
El Paso, TX						
SPCA	0	0.707	0	0.707	0	−0.023 (0.009)
PCA	0.493	0.538	0.573	0.128	0.35	−0.015 (0.01)
Houston, TX						
SPCA	0	0	0	0	0	0 (0)
PCA	0.281	0.56	0.516	0.354	0.465	0.002 (0.008)
Jersey City, NJ						
SPCA	0.707	0	0	0.707	0	−0.006 (0.015)
PCA	0.487	0.522	0.502	0.046	0.485	−0.004 (0.012)
Nashville, TN						
SPCA	0.587	0.568	0.552	0	0.168	−0.023 (0.010)
PCA	0.606	0.542	0.432	0.380	0.094	−0.023 (0.011)
Pittsburgh, PA						
SPCA	1	0	0	0	0	0.005 (0.003)
PCA	0.512	0.53	0.486	0.196	0.427	0 (0.004)
Salt Lake City, UT						
SPCA	0	0	0	0	0	0 (0)
PCA	0.415	0.54	0.539	−0.105	0.484	−0.022 (0.012)

a The loadings given to each pollutant by SPCA and PCA. A loading of 0 for SPCA means that the pollutant was not included in the subset of pollutants retained by SPCA.

b The estimated increase in mortality (± SE) for a simultaneous 1 SD increment in the concentration of each pollutant. 100× this value is approximately the percentage increase in mortality. SEs of these estimated effects are in parentheses.

**Table 5:** Pairwise correlations between the mortality (Mort), temperature (Temp), dewpoint temperature (Dew), and pollutant time-series data for Cleveland, OH, and Nashville, TN.

	Mort^a^	Temp^b^	Dew^c^	PM_10_	O_3_	NO_2_	SO_2_	CO
Cleveland, OH								
Mort	1	−0.07	−0.07	0.04	−0.02	0.02	0.02	0.03
Temp	−0.07	1	0.91	0.41	0.66	0.09	0.04	0.02
Dew	−0.07	0.91	1	0.36	0.52	0.07	−0.02	0.04
PM_10_	0.04	0.41	0.36	1	0.56	0.63	0.48	0.48
O_3_	−0.02	0.66	0.52	0.56	1	0.36	0.26	0.21
NO_2_	0.02	0.09	0.07	0.63	0.36	1	0.56	0.67
SO_2_	0.02	0.04	−0.02	0.48	0.26	0.56	1	0.4
CO	0.03	0.02	0.04	0.48	0.21	0.67	0.40	1
Nashville, TN								
Mort	1	−0.19	−0.18	−0.07	−0.11	−0.04	0.03	0.01
Temp	−0.19	1	0.94	0.33	0.69	0.11	−0.23	−0.22
Dew	−0.18	0.94	1	0.31	0.56	0.08	−0.24	−0.22
PM_10_	−0.07	0.33	0.31	1	0.45	0.44	0.08	0.40
O_3_	−0.11	0.69	0.56	0.45	1	0.26	−0.1	−0.07
NO_2_	−0.04	0.11	0.08	0.44	0.26	1	0.08	0.36
SO_2_	0.03	−0.23	−0.24	0.08	−0.10	0.08	1	0.08
CO	0.01	−0.22	−0.22	0.4	−0.07	0.36	0.08	1

a Nonaccidental daily deaths of individuals ≥ 65 years of age.

b The current day’s mean 24-hr temperature.

c The current day’s mean 24-hr dew point temperature.
